# Effects of salbutamol and phlorizin on acute pulmonary inflammation and disease severity in experimental sepsis

**DOI:** 10.1371/journal.pone.0222575

**Published:** 2019-09-19

**Authors:** Léia Cardoso-Sousa, Emilia Maria Gomes Aguiar, Douglas Carvalho Caixeta, Danielle Diniz Vilela, Danilo Pereira da Costa, Tamires Lopes Silva, Thúlio Marquez Cunha, Paulo Rogério Faria, Foued Salmen Espindola, Ana Carolina Jardim, Alexandre Antônio Vieira, Tales Lyra Oliveira, Luiz Ricardo Goulart, Robinson Sabino-Silva

**Affiliations:** 1 Department of Physiology, Institute of Biomedical Sciences, Federal University of Uberlandia, Minas Gerais, Brazil; 2 Institute of Biotechnology, Federal University of Uberlandia, Minas Gerais, Brazil; 3 Institute of Biomedical Sciences, Laboratory of Immunoparasitology "Dr. Mario Endsfeldz Camargo", Federal University of Uberlandia, Minas Gerais, Brazil; 4 Department of Pulmonology, School of Medicine, Federal University of Uberlandia, Minas Gerais, Brazil; 5 Department of Morphology, Institute of Biomedical Sciences, Federal University of Uberlandia, Minas Gerais, Brazil; 6 Laboratory of Virology, Institute of Biomedical Sciences, Federal University of Uberlandia, Minas Gerais, Brazil; 7 Faculty of Medicine, Municipal University of Sao Caetano do Sul, Sao Paulo, Brazil; 8 Department of Medical Microbiology and Immunology, University of California Davis, California, United States of America; National Institutes of Health, UNITED STATES

## Abstract

Respiratory infection can be exacerbated by the high glucose concentration in the airway surface liquid (ASL). We investigated the effects of salbutamol and phlorizin on the pulmonary function, oxidative stress levels and SGLT1 activity in lung, pulmonary histopathological damages and survival rates of rats with sepsis. Sepsis was induced by cecal ligation and puncture surgery (CLP). Twenty-four hours after surgery, CLP rats were intranasally treated with saline, salbutamol or phlorizin. After 2 hours, animals were anesthetized and sacrificed. Sepsis promoted atelectasis and bronchial inflammation, and led to increased expression of SGLT1 on cytoplasm of pneumocytes. Salbutamol treatment reduced bronchial inflammation and promoted hyperinsuflation in CLP rats. The interferon-ɤ and Interleucin-1β concentrations in bronchoalveolar lavage (BAL) were closely related to the bronchial inflammation regulation. Salbutamol stimulated SGLT1 in plasma membrane; whereas, phlorizin promoted the increase of SGLT1 in cytoplasm. Phlorizin reduced catalase activity and induced a significant decrease in the survival rate of CLP rats. Taken together, sepsis promoted atelectasis and lung inflammation, which can be associated with SGLT1 inhibition. The loss of function of SGLT1 by phlorizin are related to the augmented disease severity, increased atelectasis, bronchial inflammation and a significant reduction of survival rate of CLP rats. Alternatively salbutamol reduced BAL inflammatory cytokines, bronchial inflammation, atelectasis, and airway damage in sepsis. These data suggest that this selective β2-adrenergic agonist may protect lung of septic acute effects.

## Introduction

Sepsis is a serious clinical condition that represents a response to a severe infection that may lead to multiple organs damage and acute lung injury [1]. In severe sepsis a poor prognosis with high mortality rates is achieved when one or more organs are affected [2, 3]. The lung is the most vulnerable organ during sepsis. The acute respiratory distress syndrome (ARDS) occurs in 25% to 50% of patients with sepsis [4]. Considering the high mortality rate in patients with ARDS, the identification of therapeutic platforms that are innovative, safe and effective are crucial for successful sepsis treatment [5].

Severe sepsis induces massive infiltration of inflammatory cells and an increase of thiobarbituric acid reactive substances (TBARS) levels in lung. Caspase-mediated oxidative stress leads to lung apoptosis in severe lung injury by sepsis [6]. Sepsis, among other complications, promotes ventilatory dysfunction [7] as well as impairment on the cardiorespiratory responses to chemoreflex activation in awake rats [8]. These changes are associated with the increase of inflammatory cytokines in bronchoalveolar lavage (BAL) as interferon (IFN)-gamma, Interleucin-1β, interleukin-6 and tumor necrosis factor alpha (TNF-alpha) [9], and its inflammatory balance is related to the severity and mortality of murine sepsis [9].

The β-2 adrenergic agonists have been described as efficient treatment for airway inflammation. Salbutamol activates β-2 adrenergic receptors and inhibits inflammatory genes in several tissues [10]. The stimulation of SGLT1-mediated sodium-dependent glucose transport activity in type I and type II pneumocytes with isoproterenol, a non-selective beta-adrenergic agonist, reduced glucose concentration in airway surface liquid (ASL). Glucose is removed from ASL through SGLT1-mediated secondary active sodium-coupled transport, suggesting an imperative role of SGLT1 protein in bacterial proliferation [11]. However, despite the clear demonstration of cAMP-PKA pathway activation through enhancement of SGLT1-mediated glucose transport, the capacity of salbutamol (a selective β2-adrenergic agonist) to modulate SGLT1 protein has never been investigated in lung and other tissues.

In contrast, the intranasal administration of phlorizin, an inhibitor of SGLT1 cotransporters, increased glucose concentration in ASL under normoglycemic and hyperglycemic conditions [11]. Although the relationship between phlorizin effects and bacterial proliferation was well characterized [11], the phlorizin effects in lung to modulate histopathological damages, oxidative stress and survival rate were not considered in sepsis. Thus, the aims of the present study were to investigate the lungs of rats with sepsis under intranasal treatments with phlorizin or salbutamol, mainly observing: i) histopathological damages; ii) inflammatory cytokines in BAL; iii) oxidative stress; iv) the SGLT1 protein localization in alveolar cells; and v) acute survival rate.

## Methods

### Animal procedures

The research staff received special training in animal care or handling by CBEA/PROPP/UFU. All animal procedures were approved by the Ethics Committee for Animal Research of the Federal University of Uberlandia (Approval No. 45/2015).

The cecal ligation and puncture surgery (CLP) was performed in male Wistar rats (weighing ~ 260g) to trigger sepsis-induced lung injury. To CLP procedures, rats were anesthetized by an intraperitoneal injection of ketamine (90 mg/kg) and xylasine (10 mg/kg). Rats underwent an aseptic midline laparotomy, and the portion of the cecum was exteriorized and placed outside of the abdominal cavity. The cecum was partially ligated using a 4.0 silk tie, perforated nine times with a 22-gauge needle and then gently squeezed to extrude a small amount of feces from the perforation. The cecum was returned into the abdominal cavity and the laparotomy was closed using a 4.0 silk sutures. Sham animals underwent the same procedure; however, cecum was not ligated and perforated [9]. Animals were caged and allowed free access to water and standard rodent chow diet (Nuvilab CR-1; Nuvital, Curitiba, Brazil). The animals were observed for twenty-four hours after surgery. Animals were kept in dorsal recumbence throughout the experimental procedures. Body temperature was maintained at 37.5 ± 1.5°C with a heating blanket. Sham and CLP rats were studied 24 hours after sepsis induction.

To demonstrate the effects of β2-adrenergic agonist, CLP rats received salbutamol (0.15 mg/kg; 100 μL; CLP-salb). For inhibition of SGLT1 activity, CLP rats were submitted to phlorizin (10^-3^ M; 100 μL; CLP-phlo), whereas saline 0.9% was used as a control (vehicle; 100 μL; CLP-sal). Sham rats received saline 0.9% (vehicle; 100 μL; Sham). All treatments were performed intranasally two hours before sample collection, using a micropipette, and under anesthesia (ketamine (90 mg/kg) and xylasine (10 mg/kg), intraperitoneally). Bronchoalveolar lavage and lungs were obtained from anesthetized rats. All efforts were made to minimize animal suffering.

The methods were carried out in accordance with the approved guidelines. We considered humane endpoint with specific criteria: 1) in the cases of ruffled fur and ocular discharge is indicated careful monitoring; and 2) in cases of ataxia, tremor and cyanosis is indicated euthanasia. However, probably due to the short period of sepsis, it was not necessary to perform euthanasia in animals.

### Bronchoalveolar lavage (BAL) collection

Under anesthesia, the trachea was accessed, and a 19-gauge needle was gently inserted into the trachea. One milliliter of physiological saline was slowly injected, and gentle aspirations were performed to collect ~300 μL of bronchoalveolar lavage (BAL). The BAL was immediately stored in nitrogen and later at -20°C for further analysis. For the inflammatory cytokines evaluation, BAL was centrifuged at 1,000 rpm at 4 °C. Finally, the left ventricle was sectioned, lungs were exsanguinated and collected for further analyses.

### Histopathological analysis of lung

Histopathological analyses of lung samples were performed by hematoxylin-eosin staining. The trachea was clamped at end-expiration of bronchoalveolar lavage (BAL). Lungs were removed *en bloc* and abdominal aorta and vena cava were sectioned to quickly kill the animal by exsanguination. To preserve pulmonary architecture, the lower and middle lobes of the right lung were fixed with 4% formaldehyde in phosphate buffer (PB) prior to paraffin inclusion. Lung sections were deparaffinized in xylol/xylene and rehydrated with a graded series of ethanol. To evaluate histopathological patterns, tissues were cut into 5-μm sections for hematoxylin-eosin (HE) [11]. Lungs were examined by light microscope (Leica ICC50). Photomicrographs at magnifications of x400 were obtained from non-overlapping fields per section. Airway damage was quantified using a scoring system protocol [12]. Briefly, scores of 0 to 4 were used to represent the severity of atelectasis and bronchial inflammation with 0 standing for no effect and 4 for maximum severity effect. To represent scores of inflation, score 1 was used to hypoinsuflation, score 2 to normoinsuflation and score 3 to hyperinsuflation. Hyperinflation was characterized by large-volume gas-exchanging air spaces (structures with morphology distinct associated with alveoli higher than 120 μm) [12, 13]. Furthermore, the extent of each scored characteristic per field was stated with a scale between 0 to 4, with 0 standing for no visible evidence and 4 for complete involvement per field (quadrants). Specific scores to atelectasis, bronchial inflammation and inflation were calculated as the product of severity and extent of each feature. The cumulative airway damage score was calculated as the sum of each score characteristic (atelectasis, bronchial inflammation and inflation).

### Survival rate

To analyze the effect of salbutamol and phlorizin on the survival rates of septic rats, were recorded every hour until the endpoint at 26 h.

### Immunofluorescence analysis

The high lobe of the right lung was fixed in 4% formaldehyde phosphate buffer (PB) followed by cryoprotection in increasing sucrose solutions (10%, 20% and 30%) in PB. Seven-μm-thick sections were placed on gelatin-coated slides (Sigma Chem. Co., St Louis, USA), and subjected to immunodetection using anti-rat SGLT1 antibody (1:100, Merck Milipore, Germany, catalog number 07–1417), followed by incubation with Alexa Fluor 488 (1:150, Molecular Probes, Eugene, Oregon, USA, catalog number A21441). F-actin staining was performed with rhodamin-phalloidin (1:200, Molecular Probes, Merck Milipore, Germany, catalog number R415). After washings, tissue sections were coverslipped and analyzed in LSM 510 Meta laser-scanning confocal microscope (Carl Zeiss). The level of SGLT1 protein in the plasma membrane or in the cytoplasm was quantified using ImageJ software (ImageJ Software, National Institutes of Health, Bethesda. MD, USA).

### Oxidative stress marker analysis

Thiobarbituric acid reactive substances (TBARS) were measured in lower lobes of the right lung by reacting with malondialdehyde (MDA) and thiobarbituric acid (0.67% TBA). The organic phase was evaluated with a fluorometer at 515 nm excitation and 553 nm emission. MDA standard curve allowed quantification of this compound in the samples by linear regression [14].

### Catalase, Superoxide Dismutase (SOD) and Glucose 6-Phosphate Dehydrogenase Activity (G6PDH) antioxidants activity

The catalase activity of lung tissues was assayed as described previously [15]. Briefly, lung homogenate was added with H_2_O_2_ in 50 mM KP buffer (pH 7.0), and H_2_O_2_ decomposition was monitored at 420 nm. One unit of catalase activity catalyzed the degradation of 1 μmol of H_2_O_2_ per min. Activity of SOD was measured by the inhibition of autoxidation of pyrogallol. This inhibition occurs in the presence of SOD and was evaluated using a spectrophotometer at 420 nm. A calibration curve was constructed using SOD as standard. A 50% inhibition of pyrogallol was defined as one SOD unit (U). The results were calculated as U/mg of protein. Glucose 6-phosphate dehydrogenase was monitored by the production of NADPH with a consequent increase in absorbance at 340 nm. Readings were performed in microplates, where the samples were incubated with Tris-HCl buffer (100 mM, pH 7.5), magnesium chloride (MgCl2 2 M), NADP + (0.5 mM), and glucose 6-phosphate (1 mM) [14].

### Protein and pro-inflammatory cytokines concentration in BAL

Protein concentration was performed by the Bradford colorimetric method based on the formation of complexes between the dye Coomassie Brilliant Blue with the polypeptide chain. Five microliters of lung extracts or BAL were placed in a microplate. Absorbance was obtained with a GENESYS 10S UV-VIS spectrophotometer in a wavelength of 595nm. Enzyme-linked immunosorbent assay (ELISA) kits were used to measure interferon-ɤ and Interleucin-1β (BD Biosciences, San Jose, CA, USA) in BAL, according to the manufacturer’s protocol. Results were interpolated from a standard curve using recombinant cytokines (pg/mL).

### Statistical analysis

All values are reported as mean ± SEM. Number of animals is described in legends. Comparisons of the means were performed by one-way analysis of variance (ANOVA), followed by mean comparisons through the Student-Newman-Keuls post-test (GraphPad Prism version 7.00 for Windows, GraphPad Software, San Diego, CA, USA). The correlation between mean values of catalase activity and tidal volume per body weight was analyzed by the Pearson correlation test. Student´s t-tests were performed as appropriate. Survival rates were analyzed by Chi-square using log-rank (Mantel-Cox) test. Values of *P* < 0.05 were considered as statistically significant.

## Results

The CLP rats were acutely treated with saline (sal), salbutamol (salb), or phlorizin (phlo); thus, the following groups were studied: Sham, CLP-sal, CLP-salb and CLP-phlo.

The twenty-four-hour CLP-sal, CLP-salb and CLP-phlo animals did not demonstrated change body weight (*P*> 0.05; data not shown).

### Effect of sepsis, salbutamol and phlorizin in oxidative stress in lungs

**Fig 1:**
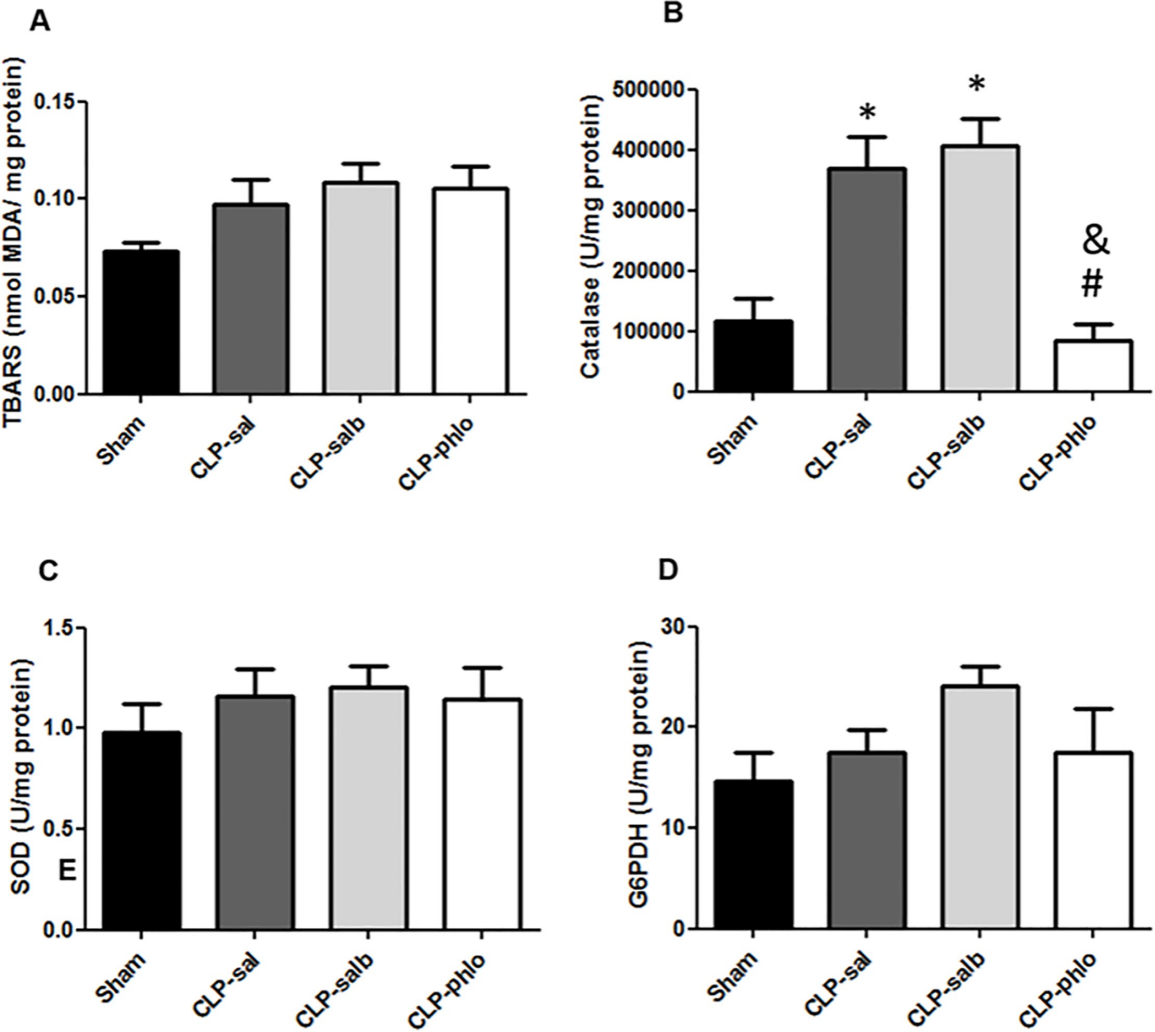
Oxidative stress in lungs. Lipid peroxidation- thiobarbituric acid reactive substances (TBARS) (A), catalase (B), Superoxide Dismutase (SOD) (C) and Glucose 6-Phosphate Dehydrogenase Activity (G6PDH) (D) were analyzed in lungs of Sham, CLP saline (CLP-sal), CLP salbutamol (CLP-salb) and CLP phlorizin (CLP-phlo) treated rats. Results are represented as mean ± SEM of 5 animals; *P <0.05 vs Sham; and #*P <*0.05 vs CLP-sal; &P < 0.05 vs CLP-salb. One-way ANOVA followed by Student-Newman-Keuls post-test.

Oxidative stress analyses in lung of rats with sepsis are shown in Fig 1. Lipid peroxidation (TBARS, Fig 1A) was unchanged in CLP-sal compared to Sham (despite the increase of ~30%, ~45% and ~45% in mean of CLP-sal, CLP-salb and CLP-phlo rats, respectively). Antioxidant defense systems were analyzed by activity of catalase (Fig 1B), Superoxide Dismutase (SOD) (Fig 1C), and Glucose 6-Phosphate Dehydrogenase Activity (G6PDH) (Fig 1D) in the lung. Catalase activity increased in CLP-sal compared to Sham rats (*P*> 0.05). No change of catalase levels were observed in CLP-salb (*P*> 0.05); however, CLP-phlo) presented a significant reduction in the levels of catalase activity (*P*< 0.05). Sepsis and treatments had no effect on SOD (Fig 1C) and G6PDH (Fig 1D) activities in the lung.

### Effect of sepsis, salbutamol and phlorizin on subcellular distribution of SGLT1 protein in the rat lung

**Fig 2:**
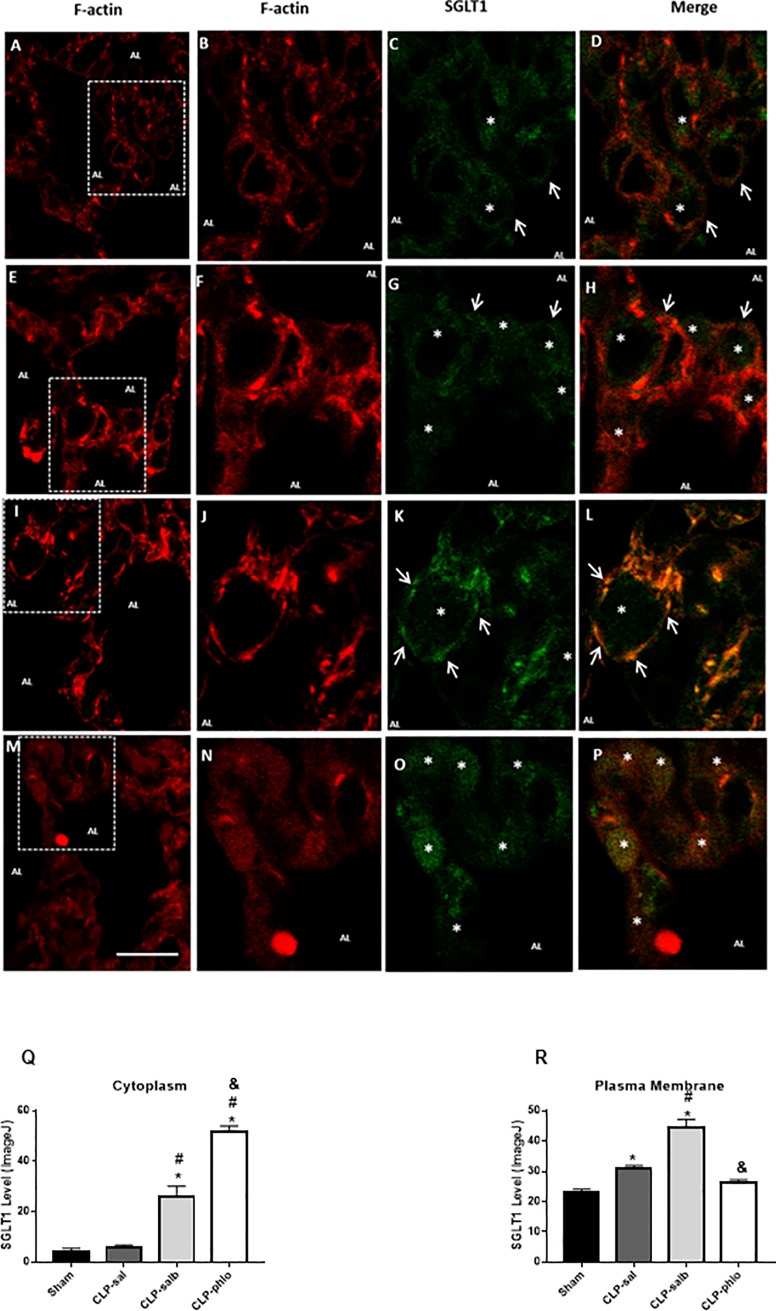
SGLT1 staining in lung tissues. Alveolar structures in lung from Sham (A to D), CLP saline (CLP-sal, E to H), CLP salbutamol (CLP-salb, I to L) and CLP phlorizin (CLP-phlo, M to P) treated rats. Sections (A, E, I and M) were immunostained with anti-F-actin antibody (red). White enclosed boxes showing an alveolar septum and alveolar lumen, taken with a greater resolution, are presented in the next sections: (B, F, J and N) F-Actin (red), (C, G, K and O) SGLT1 (green) and (D, H, L and P) merged photomicrographs for co-localization of SGLT1 and F-actin (yellow to orange). White arrows indicate the presence of SGLT1 in the luminal membrane. White asterisks indicate SGLT1 in cytoplasm. Description: (AL) alveolar lumen of the rat pulmonary section. Magnification, x600, scale bar, 10 μm. Section Q and R show the protein level in cytoplasm or plasma membrane, respectively. Images are representative of 4 animals in each group.

Immunodetection of F-actin (red color) and SGLT1 (green color) in pulmonary alveoli is shown in Fig 2. This figure shows the F-actin immunodetection in pulmonary alveoli of sham, CLP-sal, CLP-salb and CLP-phlo rats (Fig 2A, 2E, 2I and 2M; respectively), from which the squared marked alveolar septum was amplified and analyzed for F-actin (Fig 2B, 2F, 2J and 2N) and SGLT1 (Fig 2C, 2G, 2K and 2O), as well as for the merged image (orange to yellow colors, Fig 2D, 2H, 2L and 2P). In lung alveolar cells of sham rats (Fig 2A to 2D), SGLT1 protein expression was detected in luminal membrane and in the intracellular region (cytoplasm as a reserve pool). Sepsis promoted an increase of SGLT1 protein in the intracellular region, despite the presence of SGLT1 in plasma membrane. The intracellular presence of SGLT1 in alveolar cells of lung from CLP-sal rats was detected in central part of the cell and near the plasma membrane (Fig 2E to 2H). The β2-adrenergic agonist salbutamol (Fig 2I to 2L) promoted a strong enhanced expression of SGLT1 content in luminal membrane of alveolar cells. The co-expression of SGLT1 and F-actin reinforced the salbutamol-induced SGLT1 translocation, as evinced by the yellow color. Phlorizin treatment blocked the SGLT1 staining in plasma membrane, suggesting effective inhibition of SGLT1 function, which was associated with SGLT1 detection mainly in the intracellular region. The quantified level of SGLT1 protein in the plasma membrane or in the cytoplasm corroborate these data (Fig 2Q to 2R).

### Effects of CLP and salbutamol on alveolar and bronchiolar structures in rats

**Fig 3:**
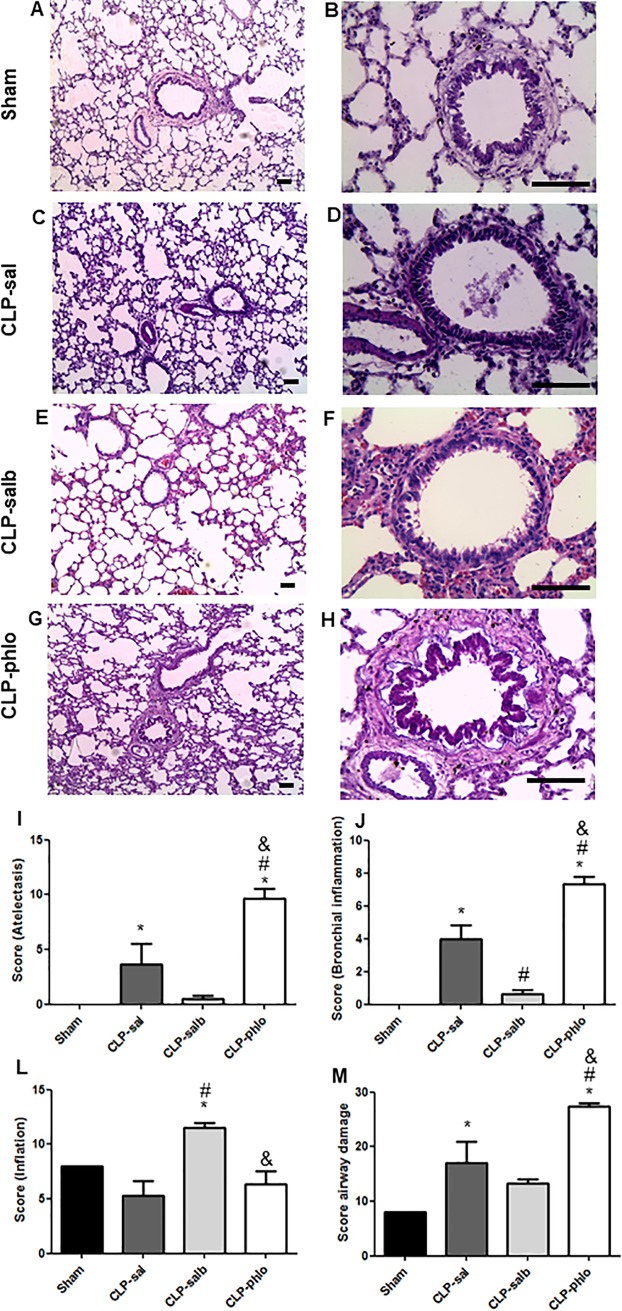
Analyses of lung tissue structure by hematoxylin-eosin staining. Alveolar and bronchiolar structures in lung from Sham, CLP saline (CLP-sal), CLP salbutamol (CLP-salb) and CLP phlorizin (CLP-phlo) treated rats. Hematoxylin-eosin stained sections (A-H), Sham (A-B), CLP-sal (C-D), CLP-salb (E-F) and CLP-phlo (G-H) magnification, x400, and severity of atelectasis (I), bronchial inflammation (J), inflation (L) and airway damage scores (M); Images are representative of 4–5 animals in each group. Scale bar, 70 μm. *P <0.05 vs Sham; and #*P <*0.05 vs CLP-sal; &P < 0.05 vs CLP-salb. One-way ANOVA followed by Student-Newman-Keuls post-test.

To determine whether sepsis, salbutamol and phlorizin promoted airway damage in lungs, lung sections were stained with hematoxylin-eosin (Fig 3A–3H). Scores of atelectasis (Fig 3I), bronchial inflammation (Fig 3J), inflation (Fig 3L) and airway damage (Fig 3M) were also showed in Fig 3. The alveolar and bronchiolar structures remained unaltered in Sham rats without atelectasis and bronchial inflammation. Besides, CLP increased scores of atelectasis (P < 0.05) and bronchiolar inflammation (P<0.05), but not changed scores of inflation (P > 0.05). Salbutamol treatment reduced scores of bronchial inflammation (P<0.05) and increased score of inflation (P<0.05). Besides, salbutamol treatment did not promote changes in the scores of atelectasis in CLP-sal rats (P > 0.05). Phlorizin treatment increased scores of atelectasis and bronchial inflammation compared with CLP-sal rats (P < 0.05).

### Effect of salbutamol or phlorizin on BAL protein and pro-inflammatory cytokines concentration of rats with sepsis

**Fig 4:**
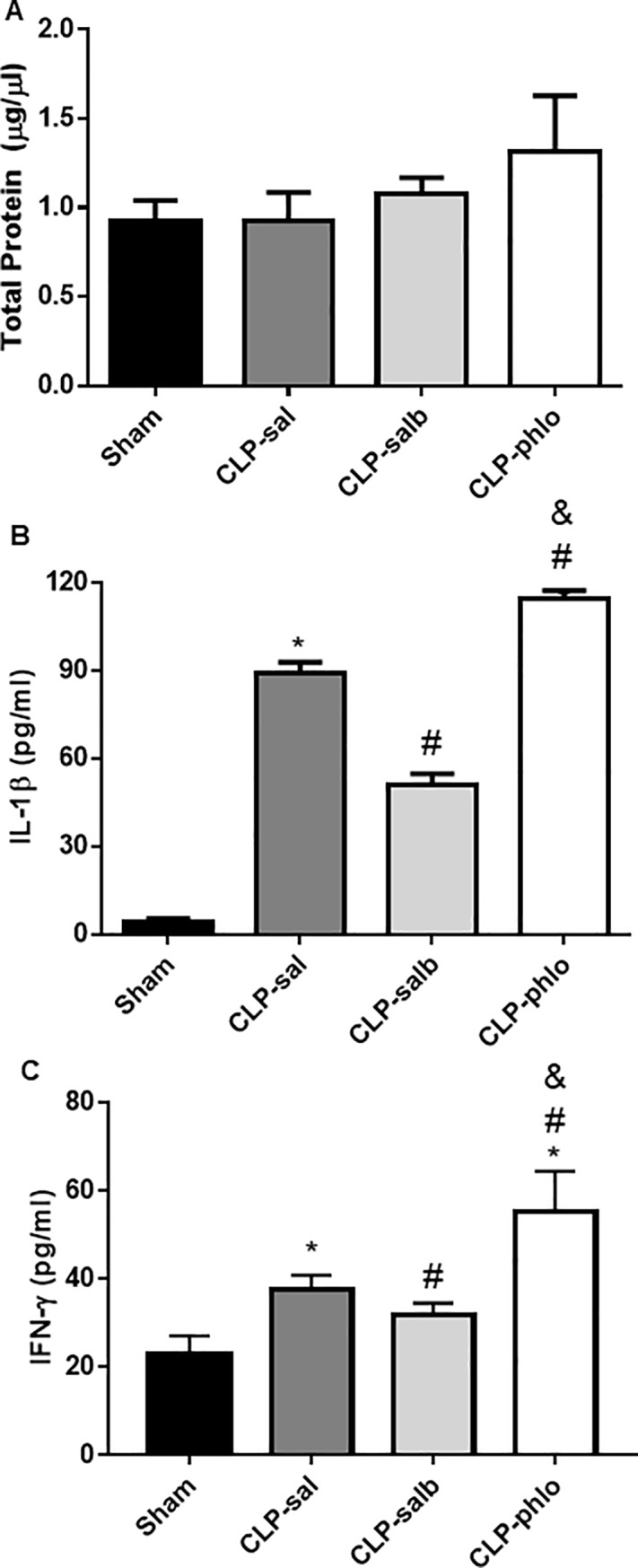
Total protein concentration and pro-inflammatory cytokines in bronchoalveolar lavage. **(**A) Total protein concentration, (B) interleucin-1β and (C) interferon-ɤ was analyzed in BAL from Sham, CLP saline (CLP-sal), CLP salbutamol (CLP-salb) and CLP phlorizin (CLP-phlo) treated rats. Results are mean ± SEM of 5–6 animals; *P <0.05 vs Sham; and #*P <*0.05 vs CLP-sal; &P < 0.05 vs CLP-salb; One-way ANOVA followed by Student-Newman-Keuls post-test.

Fig 4 shows total protein concentration measured in BAL. Sepsis, salbutamol and phlorizin did not change (*P* > 0.05) the BAL total protein concentration in rats. CLP increased levels of BAL interleucin-1β and interferon-ɤ than Sham rats (P < 0.05). Besides, salbutamol treatment reduced levels of these pro-inflammatory cytokines (P < 0.05) and, alternatively, phlorizin treatment increased scores of BAL interleucin-1β and interferon-ɤ than CLP-sal rats (P < 0.05).

### Effect of phlorizin on survival rate in septic rats

**Fig 5:**
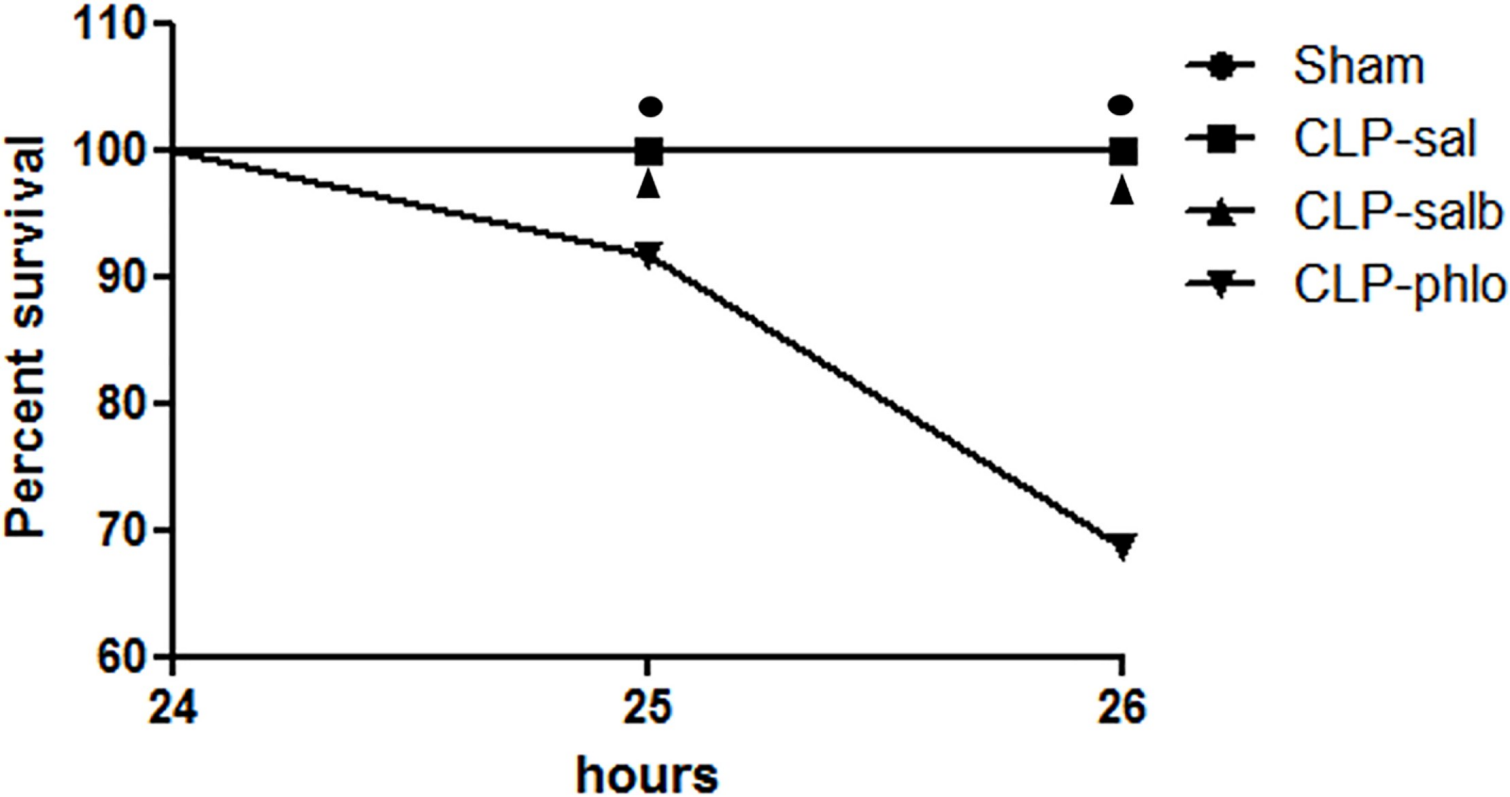
Survival analyses. Kaplan-Meier curve was constructed with survival rates of CLP saline (CLP-sal), CLP salbutamol (CLP-salb) and CLP phlorizin (CLP-phlo) treated rats. Results are represented by mean values of 7–12 animals per group; *P <0.05 vs Sham, CLP-sal and CLP-salb. Chi square = 9,167 using log-rank (Mantel-Cox) test.

As demonstrated in Fig 5, the survival rates of Sham, CLP-sal and CLP-salb rats were 100% 24h after CLP or simulated surgery. Phlorizin treatment demonstrated to markedly decrease the survival rates in 1-hour (25 h) and 2-hours (26 h) animals 24 following CLP (P < 0.05). The Log-rank (Mantel-Cox) test showed Chi-square of 9.167 for phlorizin treated CLP rats (vs. Sham, CLP-sal and CLP-phlo).

## Discussion

The present study indicates that sepsis induces atelectasis, bronchial inflammation and airway damage. The intranasal salbutamol treatment of lungs in septic rats showed increased of the SGLT1 expression in luminal membrane of lung alveolar cells, reduction of atelectasis, bronchial inflammation and reduction of airway damage, suggesting a potential benefit in ARDS secondary to sepsis. Besides, our data indicate that intranasal phlorizin treatment, a classical SGLT cotransporters inhibitor, increased the severity of atelectasis and bronchial inflammation and inhibited the antioxidant system in lungs of septic rats. Finally, our study unravels that salbutamol-treated septic patients might restore airway homeostasis by reducing atelectasis and lowering bronchial inflammation. Besides, the therapy of dual SGLTs inhibitors are at high risk of pulmonary damage.

Sepsis can be experimentally induced by a procedure known as CLP, which mimic human sepsis [16,17]. Some evidences have reported that acute lung damage secondary to sepsis increases oxidative stress (MDA contents, reactive oxygen species production) and inflammation [1, 18]. However, other studies revealed normal levels of oxidative stress in acute respiratory distress secondary to sepsis [19, 20]. The increased of oxidative stress can stimulate peroxidase-glutathione and glutathione reductase systems in several territories [21], suggesting that its increase in the antioxidant system can block the oxidative enzymes. In the present study, sepsis did not cause changes in the balance (measured by FRAP) between reactive oxygen species (ROS) and antioxidants. However, sepsis promoted increase of catalase activity in lung tissue, indicating that balance can be maintained due to high levels of this antioxidant enzyme. Absence of oxidative/antioxidative effect in the salbutamol treatment was also evidenced. Other study using CLP rats showed a differential regulation of 8-iso prostaglandin F2alpha (8-ISO) and superoxide dismutase (SOD), which was corrected by salbutamol treatment [22]. Although these results are different from our present data, further evidences of catalase inhibition by phlorizin suggested that the reasons for this discrepancy of the salbutamol treatment may be due to the different dose, route of administration, and period of observation after sepsis induction. Another important difference between our data and those reported elsewhere [22] is the type of biochemical investigation to measure the tissue oxidative stress (8-ISO vs. FRAP). Nevertheless, phlorizin reduced catalase activity in lung of rats with sepsis, suggesting an additional risk for lung damage development promoted by the oxidative stress under direct or indirect effects of the inhibitor of SGLT1 co-transporter. To our knowledge, this is a pioneer study showing oxidative/antioxidative effects of SGLT1 inhibitors in lung.

The correlation of SGLT1 function with ASL glucose concentration and microbial proliferation is well established in the literature [11]. High levels of SGLT1 in plasma membrane of alveolar cells by isoproterenol decreases ASL glucose concentration and microbial proliferation; and conversely, depletion of SGLT1 in plasma membrane by phlorizin increases ASL glucose concentration and microbial proliferation in the lungs of rats [11]. Apparently, the present study indicates that acute respiratory distress secondary to sepsis maintained SGLT1 protein in the plasma membrane of pulmonary alveolar cells. Considering that pro-inflammatory cytokines, such as IL-1β, could mediate the reduction of SGLT1 insertion in plasma membrane [23], we propose that unchanged SGLT1-mediated alveolar glucose uptake is sustained by the translocation of SGLT1 via sympathetic nervous system. Bearing in mind that the pro-inflammatory cytokines produced in the periphery can feedback to the brain, passing through the blood brain barrier at leaky points, such as organum vasculosum lamina terminalis (OVLT) and median eminence, the activation of inflammatory receptors in OVLT can stimulate sympathetic activity to several tissues [24]. As expected, after a 2-h of phlorizin treatment, very low levels of SGLT1 in the plasma membrane were observed, which reinforces that glucose transport blockage through SGLT1 was certainly guaranteed [11, 25].

We also demonstrated that salbutamol promoted SGLT1 translocation from the cytoplasm to plasma membrane of alveolar cells of lungs in septic condition. Although, the effects of β-adrenergic agonists on SGLT1 translocation has already been described in intestinal cells [26], and in acinar and ductal cells of salivary glands [27, 28]; the *in vivo* regulation of salbutamol in lung SGLT1 co-transporter was unknow. Regarding that, only one study showed effect of β2-adrenoreceptor agonist (terbutaline) in glucose uptake mediated by SGLT1 translocation (via cAMP-PKA pathway) in ruminal epithelium of sheep [26]. We already expected a parallel regulation of salbutamol in lungs of rats with experimental sepsis. However, it is important to highlight that the potential benefits of β-adrenoreceptor agonists may also diminish with continued use since prolonged exposure can result in tolerance [29], indicating the importance of understanding the effects of several β2-adrenoreceptor agonists in SGLT1 translocation of lungs.

Histological damage analyses indicate that sepsis induces atelectasis, bronchial inflammation and airway damage, and corroborate to the expected morphological changes in lung following CLP [30]. Besides, we showed that an SGLT1 inhibitor increased atelectasis and bronchial inflammation in rats with sepsis, which may play a pivotal role in ARDS. Moreover, it is important to highlight that the increased severity of atelectasis, bronchial inflammation and airway damage associated with decreased of the antioxidant system promoted by phlorizin can be related to the significant decrease in survival rate of CLP rats. Strengthening the results about the importance of SGLT1, we also showed that treatment with the β2-agonist salbutamol reduced pulmonary atelectasis and bronchial inflammation. These data are in agreement to another study that showed reduction in inflammation scoring in histopathological analysis and serum levels of TNF-α, IL-6, and IL-1β as a result of the administration of salbutamol in sepsis [30]. The salbutamol effect in the bronchial muscles promotes bronchodilation, thus the inflation effect in CLP-salb than CLP-sal rats was already expected. This increase of SGLT1 could be important to facilitate the influx of glucose/Na+/water into the cells, providing better conditions to fight against potential inflammation, while decreasing edema of the damaged area, and facilitating the process of breathing. Besides, the relationship between the increase in bronchial inflammation and higher pro-inflammatory cytokines (interferon-ɤ and Interleucin-1β) levels in BAL with decrease in survival rates of phlorizin-treated septic animals are corroborated by previous data that indicates the vital role of inflammatory mediators to the severity and mortality of murine sepsis [9]. In fact, pro-inflammatory cytokines play an important role during sepsis, specially IL-1β. It was demonstrated that a shock septic state can be induced by IL-1β in rabbits [31]. Also, the defect in the IL-1β production protected mice from development of shock septic [32]. Considering the effect in the reduction of pro-inflammatory cytokines under salbutamol treatment, the administration of this β-2 adrenergic agonists could be important to potentialize the effect of antibiotics in the treatment of sepsis increasing the prognostic of disease [33]. Salbumatol also reduced the IL-1β production and other pro-inflammatory cytokines in other tissue [34]. Thus, the present findings open avenues to new treatments for this devastating condition. The present study could alert to the potential risk of several pulmonary damages in diabetic patients using dual SGLT1/SGLT2 inhibitors affected by ARDS secondary to sepsis.

A randomized, doubled-blind placebo-controlled study to examine the safety and tolerability of canagliflozin indicated upper respiratory tract infection as adverse effects reported in over 3% of patients [35]. Federal Drug Administration (FDA) has recently approved canagliflozin for use in type 2 diabetes, while directing that a clinical outcome safety trial be undertaken [36]. Dual SGLT1/SGLT2 inhibitors, such as canagliflozin [37] and LX4211 [38], have been introduced in the diabetes pharmacopeia; however, bronchial inflammation, airway damage and reduction of survival rate should be considered in patients with sepsis. It is important to emphasize that the increased risk of diabetic patients in developing sepsis represents around 25% of all sepsis patients. It is important to highlight that the lung effects of phlorizin have not been described in normoglycemic rats. Recently, in an elegant review study, dual SGLT1/SGLT2 inhibitors have been described as promising drugs for the treatment of cancer because SGLT1 and/or SGLT2 are overexpressed in various tumors, where they deliver glucose for euglycemic glycolysis [39]. The present data suggests that a second generation of dual SGLT1/SGLT2 inhibitors that do not enter to the blood circulation and specific SGLT2 inhibitors could reduce side effects due to the expression of SGLT1 in several organs.

In summary, we showed that sepsis induces atelectasis, bronchial/BAL inflammation and airway damage. Additionally, the salbutamol treatment of septic rats presented increased of the SGLT1 activity and significant reduction of BAL pro-inflammatory cytokines, bronchial inflammation, atelectasis, and airway damage, suggesting a potential benefit in ARDS secondary to sepsis. Besides, our data indicate that phlorizin increased the severity of atelectasis and bronchial/BAL inflammation, and reduced the antioxidant system in lungs of septic rats. Finally, our study unravels benefic effects of salbutamol in ARDS secondary to sepsis.
